# Emerging Biomarkers and Biotherapies for EBV-Associated Cancers

**DOI:** 10.3390/v18090980

**Published:** 2026-09-06

**Authors:** Maria C. White, Blossom Damania

**Affiliations:** 1 Lineberger Comprehensive Cancer Center, University of North Carolina at Chapel Hill, Chapel Hill, NC 27599, USA; 2 Department of Microbiology and Immunology, University of North Carolina at Chapel Hill, Chapel Hill, NC 27599, USA

**Keywords:** EBV, viral cancers, biomarkers, immunotherapy

## Abstract

Epstein–Barr virus (EBV) is an oncogenic human gammaherpesvirus that is prevalent worldwide. Although over 95% of the world’s population harbors this pathogen, the vast majority of infections are asymptomatic, largely due to immune system involvement. However, in certain situations, such as when individuals are immunocompromised, the immune system can lose control of EBV suppression, leading to oncogenesis. While standard chemotherapeutic regimens can be effective against some EBV-associated cancers, these treatments can be toxic to some patients, and disease relapse and/or drug resistance can develop. Additionally, no human vaccine currently exists for EBV. In recent years, biotherapy has become a promising avenue for the clinical management of EBV-positive cancers. As early detection is often correlated with a more positive prognosis, identification of reliable EBV biomarkers has also come into focus. In this review, we summarize advancements made over the last five years in immunotherapy use for EBV-positive cancers and in EBV biomarker identification to detect both EBV infection and biotherapy response.

## 1. Introduction

Roughly 10% of all human cancers are caused by viral infection [1], and one of these oncogenic viruses is Epstein–Barr virus (EBV). The vast majority of the adult population is EBV-positive, often acquiring infection during childhood. The primary method of EBV transmission is through saliva, and the virus is capable of infecting both epithelial cells in the oropharynx as well as naïve B cells in the oral cavity [2]. Occasionally, EBV can also infect T cells and NK cells [3,4]. Thus, EBV-positive cancers are usually of epithelial and lymphatic cell origins, although in rarer circumstances, EBV has also been associated with sarcomas, including osteosarcoma (bone) and leiomyosarcoma (smooth muscle) [5,6].

Following infection of a naïve B cell, EBV reprograms and activates the cell, commandeering it through a germinal center-like differentiation program [7]. This ability of EBV to transform cells and induce rapid cellular proliferation holds clear oncogenic potential. In turn, the infected naïve B cell becomes a true memory B cell [8], where the virus remains latent for the lifetime of the host. To maintain survival, EBV tethers its genome to host chromatin and uses cellular machinery to replicate its DNA alongside host DNA [9]. During this latent phase, only the viral gene necessary for chromatin tethering is expressed (EBNA1) alongside non-coding RNAs [10]. This transcriptional suppression aids the virus in evading immune detection throughout the lifetime of the host.

Periodically, EBV enters the lytic phase, where viral replication occurs. Viral gene expression is massively upregulated in this phase relative to latency and includes temporally controlled expression of immediate early, early, and late viral genes. Translation of these genes allows for the production of infectious viral progeny, enabling both viral transmission to other hosts as well as infection of additional naïve B cells within the host to maintain the infected cellular pool [11].

Both the innate and adaptive immune system can detect and respond to EBV infection and reactivation. To help conceal itself during latency, EBV largely stays transcriptionally silent, and thus very few viral antigens are present for the immune system to recognize. However, during the lytic phase, viral antigen is abundant. To counteract the inevitable immune response, the virus produces a plethora of proteins that dampen or ablate the host’s antiviral defenses (for comprehensive reviews on this subject, we refer the reader to [12,13,14]). Therefore, an immune system-dependent reciprocal regulation exists between EBV and its host, which can enable lifelong infection with an oncogenic virus without cancer development. However, when host immune regulation of EBV is weakened (i.e., immunosuppression), the immune system can lose control of the virus, and EBV-mediated oncogenic progression can occur.

The mechanisms of EBV-mediated oncogenesis remain under investigation, but it is likely that EBV reactivation from latency, as well as viral gene expression, contribute to this process [15,16]. Like with all cancers, other factors (i.e., environmental, genetic, dietary) also play a role in oncogenesis. As EBV pushes infected B cells through a germinal center differentiation pattern that includes somatic hypermutation, many opportunities exist for the emergence of pro-oncogenic mutations and chromosomal translocations. As EBV-driven malignancies can occur at any time, including very late in life, de novo infection is not an oncogenic event; rather, these genetic alterations likely accumulate over time, eventually leading to a cellular proliferation event unable to be controlled by the immune system, resulting in cancer development.

## 2. EBV-Associated Malignancies

As EBV is mainly epithelial and B-cell tropic, malignancies associated with EBV infection are generally carcinomas and lymphomas, respectively. EBV has four differential gene expression patterns during latency, termed latency III, latency II, latency I, and latency 0, with a lower stage corresponding to less viral gene expression. Following primary infection, EBV gene expression is gradually restricted over time, culminating in latency 0, which occurs in infected memory B cells of healthy individuals [17]. EBV-positive cancers are classified as latency I, II, or III based on the viral genes expressed (reviewed in [18]). As latency stage increases, more viral genes are expressed in the tumor. Therefore, latency III cancers occur in the context of high immunosuppression; without strong immune pressure, EBV is able to express a larger number of immunogenic proteins. Some malignancies exist strictly in a single latency stage, while others can sometimes exhibit two different latency stages.

Nasopharyngeal carcinoma (NPC; latency II) and gastric cancer (GC; latency I–II) are both solid malignancies. While over 90% of NPC is associated with EBV infection [19], about 7.5–10% of GC is EBV-positive [20,21]. Additionally, EBV is associated with both Hodgkin lymphoma (HL; latency II) and non-Hodgkin lymphoma (NHL). Types of EBV-positive NHL include Burkitt lymphoma (BL; latency I), diffuse large B-cell lymphoma (DLBCL; latency II–III), NK/T-cell lymphoma (NKTL; latency II), and post-transplant lymphoproliferative disease (PTLD; latency III). BL is a B-cell malignancy that can be either positive or negative for EBV. The most common form of BL in sub-Saharan Africa, termed endemic BL, is overwhelmingly associated with EBV infection (>95%) [22]. DLBCL, another B-cell malignancy, can also be EBV-positive or EBV-negative. Generally, EBV-positive DLBCL cases are less treatment-responsive and have significantly poorer survival outcomes relative to EBV-negative DLBCL [23]. HL is a B-cell lymphoma characterized by the presence of unique cells termed Reed–Sternberg cells, and ~40% of cases are EBV-positive [24]. NKTL is a universally EBV-positive lymphoma associated with NK cells and T cells. Although EBV normally infects naïve B cells, some data suggest that other lymphoid cells can also be permissive to infection through multiple potential mechanisms (reviewed in [25]). Finally, PTLD is a B-cell malignancy that occurs following stem cell or solid organ transplantation. PTLD can be either positive or negative for EBV, but the majority of cases are EBV-positive.

A summary of the EBV-associated malignancies discussed in this review can be found in Table 1.

First-line therapies for EBV-associated malignancies include chemotherapy (with or without rituximab), radiation therapy, and/or surgery (Table 1). The stage of disease at the time of diagnosis is also considered prior to treatment initiation. For EBV-positive NPC, radiation with or without induction chemotherapy is typically used, resulting in a five-year survival rate of ~90% for early and locally advanced disease [26]. Surgery/endoscopic resection is often the first treatment for early-stage GC regardless of EBV infection status, as the medical presentation of EBV-positive versus EBV-negative GC is similar [27]. Combination chemotherapy has widely been used for initial treatment of EBV-positive lymphomas, with mixed success. In BL, dose-intensive chemotherapy has a high success rate in children but poor outcome in adults; as such, dose-adjusted chemotherapy is now used in adult BL patients [28]. Similar to GC, EBV-associated DLBCL is treated the same as its EBV-negative counterpart. Combination chemotherapy plus the B-cell-specific anti-CD20 drug, rituximab, is the frontline therapy for DLBCL and has been efficacious [29]. NKTL is a rare but highly aggressive lymphoma mostly found in East Asia and South America. If caught in early stages, radiation therapy can be effective for NKTL [30]. EBV-positive PTLD occurs under high immunosuppression resulting from immunosuppressive drugs given after organ transplantation. First-line treatment for EBV-positive PTLD consists of decreasing or removing the immune suppressive agent(s), in hopes of a partial return of EBV-specific immune function. Success of this approach depends on multiple factors including disease stage and organ dysfunction, with a response rate of 20–80% [31,32].

While frontline therapies for EBV-associated malignancies have varying rates of success, oftentimes they fail to kill all of the existing tumor cells. These residual cancer cells can eventually lead to disease recurrence or, if they survived due to drug resistance, establish refractory disease. In the context of relapsed or refractory (R/R) disease, the prognoses become poorer, and fewer treatment options are available to patients. Clinicians have been increasingly turning to immunotherapy to help mitigate these challenges, and major efforts have been made to identify reliable biomarkers for these diseases that will enable earlier detection and better treatment monitoring. In the next two sections, we will discuss recent advances in the use of biomarkers and biotherapies to diagnose, manage, and treat EBV-positive malignancies.

## 3. Emerging Biomarkers for Diagnosing and Managing EBV-Associated Cancers

Please note that for the following sections, “pre-clinical studies” refers to testing in cell culture and/or animal models, and “clinical studies” refers to testing in humans in clinical trials (phases I–III).

### 3.1. Nasopharyngeal Carcinoma

EBV antibodies, including anti-EBNA1 and anti-EBV capsid antigen, are commonly used to diagnose NPC, although their performance can be sub-optimal. Li et al. created and screened a large peptide library in an effort to identify novel EBV antibodies that could aid in NPC diagnosis. A retrospective, case-controlled study using this peptide library revealed a promising candidate, an antibody called anti-BNLF2b (P85-Ab). P85-Ab was then tested alongside the diagnostic standard for NPC in a prospective, case-controlled study. P85-Ab outperformed the control, exhibiting higher sensitivity, specificity, and a positive predictive value [33]. This work provides a novel EBV-positive NPC diagnostic biomarker that could be used either on its own or in combination with existing anti-EBV antibodies. Interestingly, IgA against EBNA1 has also shown promise as a predictive biomarker for NPC development in Asia. Using a prospective Singaporean cohort with validation in a Chinese cohort, the authors found that anti-EBNA1 IgA levels accurately distinguished NPC cases from matched controls with 100% specificity and sensitivity up to four years before diagnosis [34]. These data suggest that IgA titers for EBNA1 could be employed for NPC screening in these geographical areas, but its use outside Asia remains to be determined.

Previously, the EBV microRNA BART8-3p (miR-BART8-3p) had been identified as an NPC biomarker associated with metastasis in EBV-positive NPC [35]. In a subsequent retrospective, single-center study, the authors tested the diagnostic and prognostic value of miR-BART8-3p, focusing specifically on early-stage NPC in endemic areas. Results showed that miR-BART8-3p outperformed the standard as a diagnostic biomarker for stage I, but not stage II, disease [36]. Additionally, high miR-BART8-3p levels were associated with more aggressive disease and decreased survival (overall, recurrence-free, and metastasis-free) compared to low miR-BART8-3p levels, regardless of disease stage. Together, these studies characterize miR-BART8-3p as a potential diagnostic and prognostic marker for NPC, especially for early-stage disease, but validation is needed in larger cohorts.

Using salivary samples, Zheng et al. previously demonstrated that methylation density at a single CpG site on EBV DNA (11,029 bp) could be used to monitor EBV-positive NPC progression. Data showed that methylation density at this CpG site decreased upon treatment success and increased upon disease relapse [37], suggesting that the EBV DNA methylation status may provide prognostic value. To validate their finding and to test the diagnostic potential of EBV DNA methylation, the authors used self-collected samples from patients who were either NPC-positive or NPC-negative. The resulting prognostic data matched those of the original study, confirming the hypothesis that the EBV genome becomes increasingly methylated during NPC progression [38]. The authors then tested if EBV DNA methylation itself could be a biomarker for NPC. As expected, the NPC-positive samples contained methylated viral DNA. However, the control patient samples, while EBV-positive, contained predominantly unmethylated viral DNA [38]. Interestingly, this unmethylated phenotype was consistent across not only healthy controls but also individuals with non-NPC cancers, including EBV-positive lymphoma. These results are striking and offer promise for successful use of viral DNA methylation as an EBV-positive NPC-specific biomarker, although its use in early-stage disease remains to be investigated.

A landmark 2019 study identified two EBV BALF2 variants that were strongly associated with the risk of developing NPC. The authors sequenced 215 EBV isolates from patients with EBV-positive cancers, including NPC, and 54 healthy donor isolates. Importantly, these samples were taken from both NPC-endemic and non-endemic regions in China. Two single-nucleotide polymorphisms (SNPs) in the BALF2 gene, 162476T>C and 163364C>T, were found to be strong risk factors for NPC [39]. Strikingly, these two BALF2 SNPs accounted for over 80% of overall NPC risk in endemic regions. As these EBV variants are only found in Asia, this study could help explain the unique geographic distribution of EBV-positive NPC, which is most common in southern China and southeast Asia. Another study used over 100 EBV genomes isolated from NPC patient biopsies to examine EBV genomic variants for prognostic potential. Two gene variants, BBLF4-L322M and BZLF1-A205S, were identified as having prognostic value. Further analysis revealed that BBLF4-L322M was independently associated with decreased overall survival [40]. BBLF4-L322M was detectable in control samples as well, suggesting that BBLF4-L322M is not an NPC-specific biomarker; rather, it is a potential survival marker within EBV-positive NPC. However, a major caveat to the use of BBLF4-L322M as a biomarker in NPC is its geographic distribution. BBLF4-L322M was not present in datasets from other areas, including Western cohorts, meaning its use is solely localized to East Asian patients. BBLF4-L322M does not have prognostic value outside of East Asia and therefore should not be used in other geographic areas. That said, prospective studies are warranted to validate BBLF4-L322M as a survival marker in EBV-positive NPC.

Radiation and chemotherapy remain the first-line treatment for NPC. When initial therapy fails, immunotherapy has been increasingly used, often in combination with first-line agents, and has shown great promise in clinical trials. Immunotherapies used in NPC include immune checkpoint inhibitors (i.e., anti-PD1, anti-CTLA4) and T-cell-based therapies (i.e., adoptive cell therapy). A systematic review and meta-analysis of EBV-positive NPC patient data revealed that, for patients undergoing treatment with immune checkpoint inhibitors, lower baseline EBV DNA levels in the plasma were correlated with increased progression-free survival [41]. The study also showed that low EBV DNA levels during treatment and high PD-L1 levels were potential biomarkers for the success of immune checkpoint therapy in NPC. However, some limitations of this study include the relatively small sample size for each marker analyzed and limited geographic diversity, as data used were mostly obtained from China. In a phase-II clinical trial, EBV-positive NPC patients who had failed first-line chemotherapy were subsequently treated with nivolumab (anti-PD-1) and ipilimumab (anti-CTLA-4), and responders were compared to non-responders (however, a control group receiving chemotherapy only was lacking in this study). Results showed that plasma EBV DNA levels correlated with the patient response, and those with a lower viral DNA burden exhibited greater progression-free survival [42]. Pre-treatment sera analysis (post-chemotherapy but pre-immunotherapy) revealed a potential prognostic biomarker within the CD8+ T-cell compartment. Responders had more PD-1+ CTLA-4- CD8+ T cells, while non-responders had more PD-1-CTLA-4+ CD8+ T cells [42]. These findings are interesting and could suggest that CD8+ T cells expressing PD-1 are the predominant cytotoxic population. However, further studies will be needed to determine if CD8+ T-cell subpopulations can accurately predict the response to immune checkpoint immunotherapy in NPC, and how anti-PD-1/anti-CTLA-4 therapy compares to other treatment regimens.

A retrospective, single-center study examined potential predictive biomarkers for response to adoptive cell therapy in advanced NPC. In this therapy, specific T cells from the patient’s tumor, called tumor infiltrating lymphocytes (TILs), were isolated. The TILs were then exponentially expanded ex vivo, infused back into the patient, and the therapeutic response was monitored. Liu et al. used these data to determine factors that potentially correlated with the TIL infusion response. Results showed a high “double-negative” TIL population (CD3+CD4-CD8-) correlated with treatment resistance, while high CD8+ TIL populations correlated with the treatment response [43], suggesting that CD8+ TILs are the main effector cells in this therapy. Finally, the tumor suppressor gene, phosphatase and tensin homolog (PTEN), was recently identified as a potential prognostic biomarker in NPC. In addition to acting as a tumor suppressor, PTEN also regulates T-cell responses, including T-cell activation [44]. Although the sample size was small (N = 6 patients per group), the authors found that exosomal PTEN secretion was upregulated in NPC patients following treatment with radiotherapy plus immunotherapy compared to no treatment, suggesting that PTEN could be associated with treatment responses. To test this, the authors treated cell-derived exosomes with radiotherapy plus chemotherapy, and then genetically depleted PTEN from a subset of these exosomes. Both sets of exosomes were then used to treat NPC-bearing mice. Results showed that treatment with PTEN-depleted exosomes resulted in a significant increase in tumor growth compared to the control, and further in vitro studies revealed that loss of PTEN inhibited CD8+ T-cell activation and function [45]. Together, these data suggest that PTEN positively mediates the treatment response in NPC by supporting tumor-specific cytotoxic T-cell function.

### 3.2. Gastric Cancer

Due to the relatively low quantity of samples available for testing, biomarker research in EBV-positive GC has been more challenging compared to NPC. The first prospective study examining the use of EBV DNA as a biomarker in GC patients was published in 2020. This trial demonstrated that plasma EBV DNA was an indicator of treatment response in GC, as levels decreased in responder patients and increased during disease progression in non-responders [46]. With this knowledge, Alberti et al. designed the first international, multicenter, prospective observational study examining the prognostic value of EBV DNA levels in plasma for GC, called the EBV PRESAGE study [47]. However, results of this study have not been published to date.

To differentiate EBV-positive from EBV-negative GC in the clinic, in situ hybridization (ISH) is used to detect an abundant EBV antigen called EBV-encoded RNA (EBER). EBER-ISH has been the gold standard for decades and plays a vital role in diagnosis, as EBV-positive GC is both symptomatically and morphologically indistinguishable from EBV-negative GC [48]. Determining the EBV infection status at diagnosis is important in part because EBV infection itself is a prognostic biomarker for GC; EBV-positive GC cases tend to have higher overall survival compared to EBV-negative cases [49]. While EBER-ISH has been invaluable, modern advances are ushering in new detection methods that enable high accuracy with fewer false-positive results. Bai et al. developed an algorithm using next-generation sequencing that could not only diagnose EBV in GC samples with 100% specificity but also predict the efficacy of immune checkpoint inhibitor therapy [50]. Furthermore, the study revealed CTLA-4, the tumor mutational burden (TMB), and SMARCA4 gene mutation as potential biomarkers for immunotherapy success in EBV-positive GC, although the sample size was small (N = 20–22). Specifically, CTLA-4-positivity was correlated with treatment resistance; high TMB was correlated with longer survival; and SMARCA4 gene mutation was correlated with a favorable treatment response [50]. Of note, the authors’ finding in EBV-positive GC of high CTLA-4 levels corresponding to a poor treatment response was also described in EBV-positive NPC [42], suggesting a commonality across EBV-positive epithelial cancers. High PD-1/PD-L1 expression in GC was also shown to correlate with a better response to immunotherapy [51], highlighting another commonality between NPC and GC. Additionally, PD-L1 expression positively correlated with overall survival (N = 159) [51], suggesting that PD-L1 could be a prognostic marker in GC.

Previous data demonstrated that EBV miRs are abundant in EBV-positive epithelial cancers but lower in EBV-positive lymphomas, and that EBV miRs promote GC metastasis (BART10-3p and BART22) [52]. Abusalah et al. sought to understand the prognostic value of EBV miRs in NPC and GC using a systematic review of publicly available datasets from China and South Korea. Their findings suggested that high levels of EBV miRs correlated with poorer survival outcomes, and identified seven EBV miRs that had potential prognostic value (BART6-5p, BART2-5p, BART7-3p, BART13-3p, BART1-5p, BART4-5p, and BART20-5p) [53]. However, the sample size was small (N = 4 studies met inclusion criteria out of the 46 studies considered), and the potential utility of these markers outside China and South Korea remains unknown.

One of the most promising emerging biomarkers for EBV-positive GC is a gene called chromatin assembly factor 1 subunit A (CHAF1A). CHAF1A was shown to be upregulated upon EBV infection in cell culture, suggesting a potential role for this gene in EBV-mediated oncogenesis [54]. Additionally, CHAF1A expression positively correlated with TMB and PD-L1 expression in patients, both markers of immunotherapy response. Furthermore, CHAF1A expression as determined by IHC staining of patient tumor tissue positively correlated with increased infiltration of immune cells to the tumor site, suggesting that CHAF1A activates an immune response against GC [54]. As CHAF1A was tested and validated using three different patient cohorts, this gene holds great promise as both a prognostic and biotherapy response marker for EBV-positive GC. Prospective studies with diverse patient populations are needed to validate these findings.

### 3.3. Hodgkin Lymphoma

As excisional biopsy is the standard of care for lymphoma diagnosis, EBV-positive HL is diagnosed by staining tumor tissue for EBV antigens. To compare tissue biopsy with blood-based detection methods, Usha at al. designed a cross-sectional study using an HL patient cohort from Bangladesh, where 67% of participants had confirmed EBV-positive disease via lymph node biopsy and EBV antigen staining. Blood was drawn from these patients as well as from healthy control donors. Plasma EBV DNA was then measured from all samples (N = 43) using quantitative PCR. Results showed that plasma EBV DNA was 83% as sensitive and 69% as specific as immunostaining, and that EBV DNA levels did not change across disease stages [55]. These data suggest that plasma EBV DNA could be a less invasive and faster alternative to tissue staining for diagnosing EBV-positive HL. Similarly, Shen et al. conducted a retrospective, multicenter study using blood collected from patients prior to treatment initiation and found whole-blood EBV DNA could predict the prognosis of HL [56]. Overall, these data suggest that blood-based detection methods can complement immunostaining for EBV diagnosis, and prospective studies evaluating the use of plasma EBV DNA as a prognostic biomarker in HL are warranted.

### 3.4. Non-Hodgkin Lymphoma—General

Similar to other EBV-positive cancers discussed thus far, plasma EBV DNA has been studied as a potential prognostic marker in EBV-positive NHL. A retrospective study found that plasma EBV DNA levels at the time of lymphoma diagnosis correlated with disease outcomes; however, the sample sizes for all tested lymphomas except DLBCL were underpowered (HL, NKTL, mantle cell lymphoma, follicular lymphoma, anaplastic large cell lymphoma, and various T-cell lymphomas) [57]. Another study suggested that plasma EBV DNA levels were not a useful biomarker in HIV-related NHL [58], findings which were corroborated by Wang et al., at least for use as a prognostic marker prior to treatment [59]. However, this retrospective study found that plasma EBV DNA was indeed useful in monitoring the response to chemotherapy in HIV-related NHL, as detectable viral DNA during treatment correlated with worse overall survival, while progressive clearance of viral DNA during treatment was correlated with a better prognosis [59]. Overall, these data suggest that plasma EBV DNA could be a useful biomarker in NHL in certain contexts.

### 3.5. Diffuse Large B-Cell Lymphoma

A retrospective study of a DLBCL-specific patient cohort (N = 84) revealed that the EBV copy number in peripheral blood mononuclear cells (PBMCs) was predictive of the prognosis, as patients with high EBV copy numbers had worse overall and progression-free survival compared to patients with lower copy numbers [60]. These data suggest that a high EBV copy number (≥10^4^ copies/mL) is a potential prognostic biomarker in DLBCL. Similar to the findings of Wang et al. in HIV-related NHL [59], Xing et al. found that EBV DNA levels were predictive of the treatment response in DLBCL. In this retrospective, single-center study (N = 58), patients with detectable levels of EBV DNA throughout treatment had the poorest outcome, while patients who cleared viral DNA during treatment had improved responses [61]. However, prospective studies are needed to validate these findings. Finally, a retrospective analysis of a Chinese patient cohort (N = 99) sought to identify novel prognostic biomarkers in EBV-positive DLBCL. Interestingly, the authors found that an anemic state correlated with poor treatment outcomes, and hemoglobin levels lower than 90 g/L predicted lower overall survival [62]. As hemoglobin levels can be determined from a simple blood test, these findings are promising, but require validation in other geographic cohorts as well as prospective analyses.

### 3.6. NK/T-Cell Lymphoma

To compare the prognostic value of EBV DNA in NKTL, Yan et al. conducted a retrospective analysis of patient data (N = 450) from both PBMCs and plasma. Results showed that both PBMC and plasma viral DNA levels were indicative of the patient prognosis, as detectable levels of both markers correlated with poorer survival [63]. However, only plasma EBV DNA levels correlated with the treatment response. Interestingly, patients with early-stage NKTL who were positive for EBV DNA in plasma following treatment had a similar prognosis as patients with late-stage disease, and relapse only occurred in patients with detectable plasma EBV DNA. Additionally, plasma EBV DNA was more sensitive and specific than both PET/CT and CT/MRI at detecting disease recurrence [63]. Similarly, another study found that plasma EBV DNA levels were associated with treatment responses in an NKTL patient cohort (N = 111). The authors found that decreased plasma EBV DNA correlated with a positive therapeutic response, while non-responders had elevated levels of viral DNA in plasma [64]. A subsequent study quantified these prognostic parameters, demonstrating that ≥500 EBV copies/mL in whole blood or plasma prior to treatment initiation was indicative of poor survival in NKTL [65]. These data support the previous findings of other groups and underscore the potential utility of plasma EBV DNA as both a prognostic marker and relapse indicator in NKTL. Indeed, a multicenter, randomized, phase-III clinical trial examined potential prognostic biomarkers in early-stage NKTL patients undergoing pegaspargase-based chemotherapy. Plasma EBV DNA was quantified prior to treatment initiation, during treatment, and following treatment completion. Results showed that high pre-treatment viral DNA was associated with a poor therapeutic response and lower progression-free survival; viral DNA positivity during treatment was associated with lower progression-free and overall survival; and viral DNA positivity post-treatment was associated with a poor therapeutic response and decreased progression-free survival [66]. These data further support the use of plasma EBV DNA as a biomarker in NKTL. This trial also identified several potential biomarkers indicative of poor outcomes in NKTL that require further investigation, including JAK3 mutation and decreased monocyte levels [66].

As previously mentioned, EBER-ISH is often used in the diagnosis of EBV-positive malignancies. However, its use as a prognostic biomarker remains largely unknown, likely because EBER staining does not typically involve quantification. Wang et al. conducted a meta-analysis of previously published NKTL patient datasets and found that an EBER positivity of ≥75% in tumor cells was significantly suggestive of poor overall survival [65]. This finding is interesting and indicates that EBER quantification could be developed as a potential new prognostic biomarker for NKTL. More data on EBER levels in NKTL and associated survival outcomes are needed in order to define a standardized quantitative threshold for EBER positivity as it relates to disease prognosis, which will aid in accuracy and reproducibility.

### 3.7. Post-Transplant Lymphoproliferative Disorder

Although PTLD can be both EBV-positive and EBV-negative, 80% of PTLD patients have EBV-positive disease. In the absence of immune surveillance, EBV-infected B cells can rapidly proliferate, and viral reactivation is thought to play a role in this process [67]. Additionally, it has long been known that increasing systemic EBV DNA is a biomarker of active or forthcoming PTLD [68]. The EBV ZEBRA protein (also called Zta or Z), encoded by the BZLF1 gene, is required for EBV to enter the lytic cycle, and can be detected in the serum of PTLD transplant patients in its soluble form (sZEBRA) [69]. Thus, sZEBRA could serve as a biomarker for EBV-positive PTLD. Lupo et al. found that sZEBRA levels above 20 ng/mL were indicative of active PTLD, and curiously showed that sZEBRA was also present in the plasma of all patients with EBV-negative PTLD [70]. However, a major limitation of this study is the small sample size (N = 4). That said, these findings could indicate a potential role of EBV in the pathogenesis of EBV-negative PTLD, and a potential utility of sZEBRA as a biomarker in both viral and non-viral PTLD. Indeed, a subsequent retrospective, case-controlled study found sZEBRA to be associated with PTLD [71], but larger sample sizes and multicenter prospective studies are needed for validation.

As with EBV-positive epithelial cancers, EBV miRs have emerged as a potential biomarker for PTLD. It was previously shown that host miRs, including miR-19 and miR-106a, were decreased in EBV-positive PTLD relative to EBV-negative disease [72], but viral miRs as potential biomarkers for PTLD were not studied. To help fill this knowledge gap, Ji et al. sequenced EBV miRs from a pediatric transplant cohort (N = 53) where patients had either PTLD or a chronic high EBV load (CHL) in the absence of PTLD. CHL can, but will not always, develop into PTLD. Thus, changes in EBV miRs in PTLD itself could be directly studied in this cohort. Sequencing results revealed two EBV miRs to be highly upregulated in PTLD relative to CHL: BHRF1-1 and BART2-5p [73]. In mice, depleting these two EBV miRs resulted in significantly smaller tumors, suggesting that EBV miR-BHRF1-1 and -BART2-5p contribute to cellular proliferation and, potentially, PTLD progression [73]. Future work addressing this hypothesis is of interest, as is testing EBV miR-BHRF1-1 and -BART2-5p as potential biomarkers in adult populations.

Finally, methylation of the EBV genome may hold prognostic value in PTLD. Borde et al. found that EBV genomes in PTLD patients were highly methylated, similar to the viral genomes present in NPC [38]. While there was no association between viral methylation and viral load, the only patient in the study with unmethylated EBV genomes maintained a high viral load throughout treatment and was a non-responder [74], suggesting that genome methylation could be a potential biomarker for chemotherapy response in PTLD. A limitation of these findings, however, is the small sample size (N = 8). Future studies with larger sample sizes will be needed to elucidate the use of EBV DNA methylation as a prognostic biomarker in PTLD.

### 3.8. Discussion

**Figure 1:**
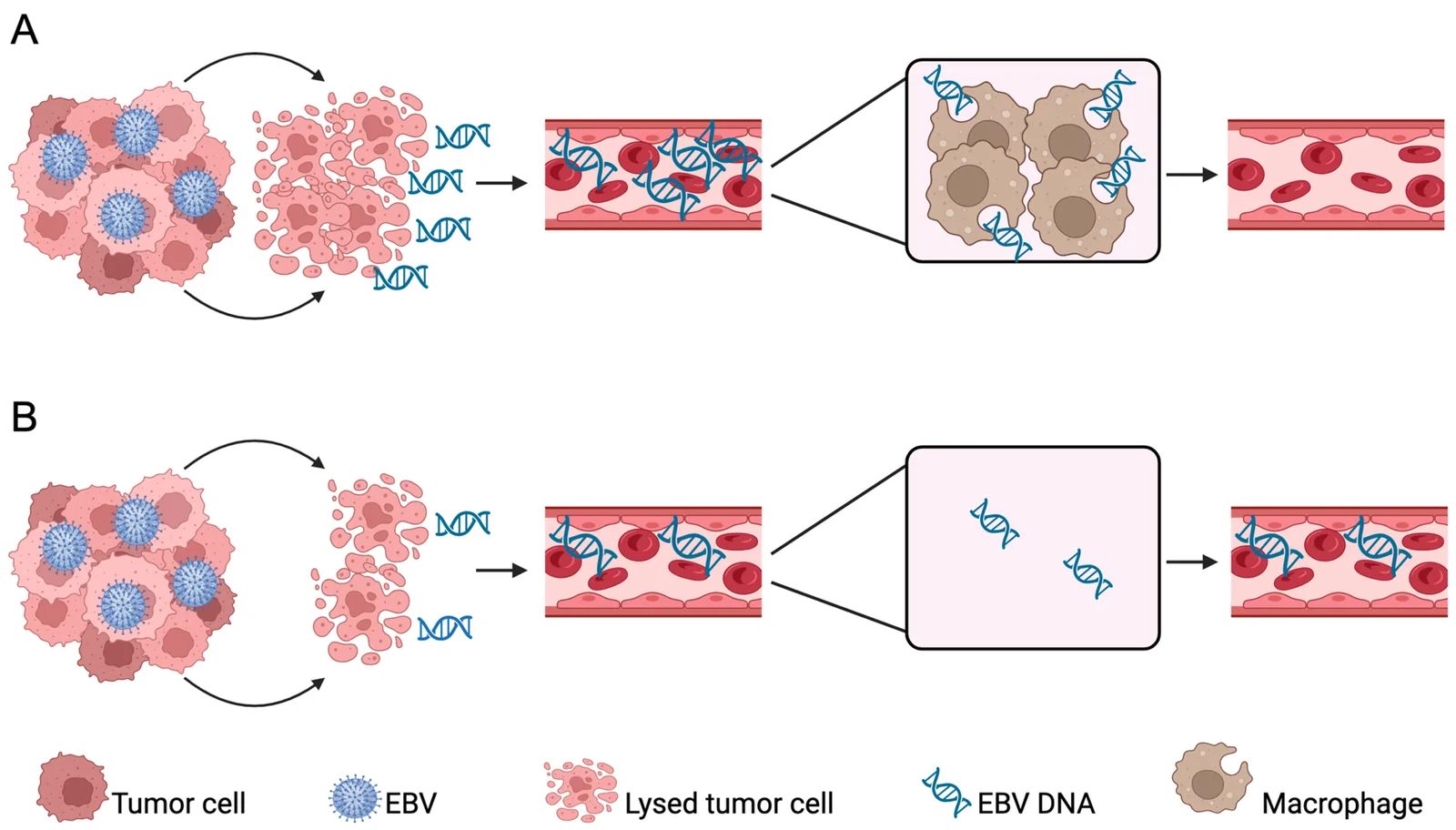
**Proposed model illustrating plasma EBV DNA as a biomarker for tumor burden and therapeutic response.** (**A**) In responder patients, robust EBV-positive tumor cell killing by the immune system and/or therapeutic intervention occurs, and viral DNA is released into the circulation. Immune surveillance cells, such as macrophages, then identify and phagocytose the viral DNA, clearing it from the bloodstream. Thus, EBV DNA is not detectable in the plasma. (**B**) In non-responder patients, a sub-optimal immune response and/or therapeutic intervention causes low to moderate EBV-positive cell death, releasing some viral DNA into the circulation. However, the absence of immune-mediated degradation (due to a weakened immune system) allows for EBV DNA to accumulate in the bloodstream, resulting in high EBV DNA levels in the plasma.

Across the EBV-associated cancers discussed, EBV DNA stands out as a common biomarker for predicting the disease outcome and response to therapy. There are several reasons why high EBV DNA levels may correlate with a poor prognosis. Perhaps EBV lytic replication contributes to sustained lymphomagenesis, which would result in more EBV DNA being released into the circulation. Another possibility is that the amount of viral DNA released systemically is directly associated with the tumor burden, assuming a high tumor burden is associated with a poor prognosis. Lastly, high EBV DNA may be an indicator of aggressive disease. A rapidly dividing tumor could quickly exhaust its blood supply/oxygen source, resulting in necrosis and subsequent release of EBV DNA into the bloodstream. The finding of high plasma EBV DNA as a marker of a poor therapeutic response is quite curious when thinking of immune system involvement. One could postulate that a strong anti-tumor immune response would result in the killing of EBV-infected tumor cells; subsequently, this immune-mediated tumor cell lysis would release EBV DNA into the circulation. In this case, elevated EBV DNA levels would reflect a more potent immune response against the cancer and, theoretically, an improved therapeutic response. Perhaps this does occur, but a strong immune response then identifies and degrades the viral DNA, thus lowering systemic levels (Figure 1A). A poor immune response, while killing fewer tumor cells, could also leave the viral DNA that is released undetected and able to accumulate in the circulation (Figure 1B). Some studies discussed above noted a progressive clearance of viral DNA during treatment in responder patients, supporting the hypothesis that EBV DNA is initially elevated due to tumor cell death, but is subsequently degraded via the immune system or some other mechanism.Further investigation into this topic would be of great interest.

A summary of the discussed biomarkers for EBV-positive malignancies can be found in Table 2. These biomarkers have potential to improve diagnosis and early detection of disease, particularly in EBV-positive NPC. Increased levels of EBNA1-IgA may identify patients at high risk of developing NPC, even years prior to diagnosis. Additionally, the presence of antibodies against the EBV protein BNLF2b (P85-Ab) may allow for earlier detection of NPC, especially near disease onset. Other markers may help inform the disease prognosis, such as EBER levels. High EBER positivity (≥75% in tumor cells) correlates with poor overall survival in NKTL, but more work is needed to confirm/establish the threshold value of positivity for prognostic use. Finally, many of these biomarkers could aid in monitoring disease progression during treatment and inform therapeutic efficacy. For example, a mutated SMARCA4 gene may be associated with a positive response to immunotherapy in EBV-GC patients, and elevated levels of the PTEN protein may also predict a favorable therapeutic response in NPC. Furthermore, anemia in DLBCL correlated with a poor response to therapy, and high CHAF1A expression may improve therapeutic responses in GC. Finally, elevated levels of CTLA-4 are associated with treatment resistance in GC, and elevated PD-1 expression is associated with immunotherapy response in both NPC and GC. Thus, biomarkers can be of value across the oncogenic spectrum, from disease risk and detection through therapeutic discussions and interventions.

## 4. Emerging Biotherapies for EBV-Positive Malignancies

### 4.1. Nasopharyngeal Carcinoma

Frontline treatment for NPC is concurrent chemoradiotherapy (CCRT), and induction (neoadjuvant) chemotherapy can also be given in some cases prior to CCRT [75]. A single-center, retrospective study examined if adding a biotherapy called tislelizumab to this regimen improved treatment efficacy and patient survival in locally advanced NPC [76]. Tislelizumab is a highly specific anti-PD-1 antibody that was previously shown to improve patient outcomes when added to standard chemotherapy in recurrent or metastatic NPC [77]. Patients received neoadjuvant chemotherapy with (N = 43) or without (N = 47) tislelizumab followed by CCRT. Results showed the addition of tislelizumab to be well-tolerated and significantly improved the complete response rate prior to CCRT. While there was no difference in overall survival between the two groups, the patients that received tislelizumab achieved significantly longer three-year progression-free survival (93%) compared to the control group (79%) [76]. Tislelizumab also increased distant metastasis-free survival. Interestingly, when stratifying based on PD-L1 expression, the survival rates for patients with high PD-L1 expression did not differ from patients with low PD-L1 expression. Results of a subsequent multicenter, double-blind, randomized, placebo-controlled phase-III clinical trial confirmed that addition of tislelizumab to existing chemotherapy conferred longer progression-free and overall survival in NPC patients compared to chemotherapy alone, including in recurrent and metastatic disease [78]. Overall, these data provide evidence supporting the use of tislelizumab in combination with chemotherapy as a frontline treatment for NPC.

Despite the widespread use of CCRT in the management of NPC, cisplatin, a common component of CCRT, can be highly toxic to patients. Additionally, a previous phase-III clinical trial in recurrent or metastatic NPC found that anti-PD-1 therapy alone achieved better results than chemotherapy [79]. Based on these findings, a multicenter phase-II clinical trial tested the use of nivolumab (an anti-PD-1 antibody) with induction chemotherapy, followed by radiotherapy (without the addition of cisplatin), followed by nivolumab monotherapy. Results showed that this treatment scheme improved failure-free survival by 10.5% compared to historical data, and patients reported a better quality of life and fewer side effects relative to CCRT. Promisingly, a multicenter, randomized, controlled phase-III clinical trial based on these findings is ongoing, with completion expected in 2027 (NCT04907370).

Although PD-1/PD-L1 blockade as a monotherapy has shown promise in NPC, the cancer can develop resistance to these antibodies, leading to recurrence, and some patients never respond to these treatments. A single-arm phase-II clinical trial enrolled NPC patients that had failed at least one line of chemotherapy and one anti-PD-1 immunotherapy and tested if a combination checkpoint blockade of both PD-1 and CTLA-4 plus chemotherapy (induction and maintenance) was efficacious in this cohort [80]. A bispecific antibody that targeted both PD-1 and CTLA-4 simultaneously, called cadonilimab, was used. Although adverse events occurred in almost all study participants (96%), the side effects were considered expected and did not linger. A partial response was observed in 56% of patients, and a complete response was observed in 12% of patients [80]. A limitation of this study was its single-arm nature; a comparison of cadonilimab plus chemotherapy with control treatment regimens was not performed. However, considering the initial refractory nature of these cancers to both chemotherapy and anti-PD-1 therapy, these results are encouraging and warrant further testing of cadonilimab plus chemotherapy for treatment of NPC that failed initial immunotherapy.

For treatment of EBV-positive diseases including NPC, therapeutic vaccination is becoming an attractive therapy in the age of mRNA vaccines. Huang et al. designed an mRNA EBV vaccine containing pre-constructed epitopes, which were shown to activate antigen-specific T cells in vivo. However, vaccination was unsuccessful at mitigating NPC disease in a humanized mouse model, despite the induced T-cell response. Therefore, the authors then tested if the addition of NK cells to the mRNA vaccine injection improved responses. Promisingly, NK-cell therapy alongside mRNA EBV vaccination resulted in significantly better tumor control compared to either therapy alone, and some animals in the combination treatment group had no detectable tumor at the end of the experiment [81]. Together, these data suggest that EBV vaccination given concurrently with NK-cell therapy synergistically reduces the NPC burden in pre-clinical studies, and offers potential promise for translation into clinical studies.

### 4.2. Gastric Cancer

While EBV-associated gastric cancer can be susceptible to immunotherapy, published results on this topic have been mixed. Al-Sattar et al. hypothesized that this could be due to disease heterogeneity involving genetic factors, environmental factors, and co-infections, leading to distinct disease subtypes within EBV-positive GC [82]. It was previously shown that GC could be grouped into “high” PD-L1 expression and “low” PD-L1 expression groups based on transcriptomic analysis. High expression of PD-L1 correlated with less aggressive disease and a better immunotherapy response compared to low PD-L1 expression [51], suggesting that immune checkpoint therapy might be more beneficial in patients with PD-L1^high^ GC.

As EBV-positive GC accounts for a tenth of all GC cases, initial clinical studies for immunotherapy use in GC tended not to stratify patients based on EBV positivity in tumor tissue. Data do suggest, however, that EBV-associated GC responds better than EBV-negative GC to treatment, including anti-PD-1 therapy [83]. Nivolumab was the first PD-1 inhibitor shown to increase overall survival in conjunction with chemotherapy in pan-GC (EBV+/−) compared to chemotherapy alone as a first-line treatment [84], paving the way for additional testing of this treatment avenue. Pembrolizumab and tislelizumab, both PD-1 inhibitors, independently provided a benefit in randomized, double-blinded phase-III clinical trials when combined with chemotherapy versus chemotherapy alone in pan-GC [85,86]. Tislelizumab and pembrolizumab are now each approved as frontline treatment with chemotherapy in unresectable or metastatic GC in the United States.

An intriguing case report described the treatment of EBV-positive GC as exhibiting high TMB and PD-L1 expression in a single patient [87]. The patient was non-responsive to chemotherapy prior to surgery, and subsequent tumor progression/invasion rendered the mass inoperable. Therefore, the patient was given chemotherapy plus nivolumab. Upon an adverse reaction to the chemotherapy, nivolumab was continued on its own. The patient responded to the immunotherapy, allowing for eventual surgical resection of the tumor. A complete pathological response was reached, and the patient went into remission [87]. Although a clear limitation of this report is its single-patient nature, these data further support the use of anti-PD-1 therapy in PD-1^high^ GC, including EBV-positive disease. Indeed, a recent single-center study examined the impact of EBV infection on nivolumab efficacy by treating one group of EBV-positive GC patients with nivolumab plus chemotherapy (N = 293) and the other group of EBV-positive GC patients with chemotherapy alone (N = 12). Results showed that the addition of nivolumab significantly improved survival outcomes (progression-free and overall) compared to chemotherapy alone in EBV-positive disease [88]. When stratified based on PD-L1 expression, however, the survival benefit was only present in the PD-L1^high^ group. Together, these data support nivolumab as a potential frontline therapy component for EBV-positive GC with high PD-1/PD-L1 expression. Additionally, a multicenter, open-label, single-arm phase-I/II clinical trial tested nivolumab plus chemotherapy as a second-line treatment in various subtypes of GC, including EBV-positive and PD-L1 positive disease. In this refractory cohort, the overall response rate was 23% [89]. However, alternative therapies are still needed for EBV-positive GC patients with low PD-1/PD-L1 expression and those whose disease does not respond to nivolumab with or without chemotherapy. Transcriptomic and immunohistological screening approaches using patient samples revealed the immune checkpoint protein, CD276, to be associated with poor outcomes in EBV-positive GC [90]. CD276, also known as B7-H3, is highly expressed on cancer cells, including cancer stem cells, and is associated with various oncogenic processes including metastasis and drug resistance [91]. Importantly, the authors found that CD276 was highly expressed in GC tissue but not in surrounding healthy tissue, suggesting that anti-CD276 could be a potential targeted therapy for EBV-positive GC. Interestingly, biopsy samples from EBV-positive GC patients who were non-responsive to nivolumab showed high CD276 expression and low CD8+ T-cell numbers, suggesting that CD276 might be decreasing the efficacy of anti-PD-1 therapy [90], although the sample size was quite small (N = 2). Nevertheless, to test this, humanized mice with EBV-positive GC were treated with an anti-CD276 compound; pembrolizumab; or a combination of both agents. Results showed that anti-CD276 therapy in combination with anti-PD-1 therapy resulted in significantly less tumor burden than either therapy alone, and that targeting CD276 in the presence of pembrolizumab restored CD8+ T-cell numbers [90]. Although this humanized mouse model used human PBMCs for immune system reconstitution and a GC cell line for tumor establishment, the authors stated that severe graft-versus-host disease was not evident in the animals, as determined by body weight. These data indicate that combination anti-CD276 therapy may restore sensitivity to anti-PD-1 therapy, but further studies will be needed to ensure that this phenotype is specific and not the result of HLA mismatching.

### 4.3. Hodgkin Lymphoma

In both adult and pediatric HL, increases in PD-L1+ cells in the tumor microenvironment have been reported [92]. Accordingly, in a multicenter, open-label, randomized phase-III clinical trial, nivolumab was shown to increase progression-free survival in combination with chemotherapy in advanced pan-HL (EBV+/−) [93] and to synergize with JAK inhibition (ruxolitinib) in an open-label phase-I clinical trial in R/R pan-HL [94]. Additionally, pembrolizumab plus chemotherapy demonstrated efficacy in previously untreated HL in a single-arm trial and multicenter phase-II clinical trial [95,96] and in R/R HL as a monotherapy following failed stem cell transplantation [97,98]. Tislelizumab, another anti-PD-1 molecule, was shown to be safe and effective in a phase-II clinical trial in R/R HL [99]. Furthermore, early results from an ongoing multicenter, non-randomized, open-label phase-II clinical trial found tislelizumab to be efficacious in newly diagnosed HL as both a monotherapy and in combination with chemotherapy [100] (NCT04843267). Together, these data underscore the effectiveness of anti-PD-1 therapy in HL, although the EBV infection status was not considered in these studies.

In pediatric EBV-positive HL, a retrospective study using 35 patient biopsies found that dual expression of PD-1 and LAG-3, another checkpoint inhibitor molecule, was associated with worse survival outcomes than single expression of either molecule. Interestingly, this phenotype was only observed in EBV-positive HL, as survival in EBV-negative pediatric HL was not affected by PD-1 or LAG-3 expression alone or together [101], suggesting that the effects of PD-1 and LAG-3 co-expression on survival are unique to viral infection and could be a potential targeted therapy for EBV-positive HL. Pre-clinical studies testing this possibility would be of interest.

Biotherapies other than immune checkpoint blockade have also shown promise in the management of HL. Although the EBV status was not a parameter in the study, a phase-II clinical trial was undertaken to determine the safety and efficacy of the bispecific antibody, AFM13, in HL. AFM13 targets both CD30 and CD16A; CD30 serves as a tumor-specific marker and is upregulated on HL cells [102], while CD16A is present on NK cells and aids in cytotoxicity [103]. By binding both tumor and NK cells at once, AFM13 brings NK cells closer to their target tumor cell and facilitates anti-tumor immunity. AFM13 was shown to be well-tolerated in patients; however, the response was modest, with a 17% response rate [104]. In effort to improve response rates, a phase-I/II clinical trial tested AFM13 in combination with NK-cell therapy (AFM13-NK) in R/R HL followed by AFM13 alone. Enrolled patients (N = 27) were previously resistant to anti-PD-1 therapy. Results were positive, with 97% of patients demonstrating a partial response and 73% achieving a complete response [105]. Follow-up results of the phase-II arm of this study are currently pending. If the EBV status of the enrolled patients is known, including a comparison of EBV-positive to EBV-negative disease outcome would be informative.

### 4.4. Burkitt Lymphoma

Bispecific antibodies have also been tested in the context of EBV-positive BL. Although the EBV protein, gp350, is a lytic protein, it is also periodically expressed on the surface of EBV-positive cancer cells during latency, and CAR-T cells targeting gp350 have shown efficacy in humanized mice bearing EBV-positive lymphomas [106]. He et al. designed a bispecific antibody that targeted both gp350 and the phagocytic marker CD89, present on neutrophils and macrophages. Testing of this antibody demonstrated that anti-gp350/CD89 lowered the viral load in immunocompromised mice and decreased tumor formation and metastasis in BL-bearing mice [107]. Thus, this study provides a potential new treatment mechanism that brings EBV-positive BL cells together with anti-tumor immune cells to facilitate tumor clearance. Additional studies are needed to help inform the utility of this therapy in EBV-positive BL.

Another biotherapy currently being tested in EBV-positive BL is CAR-T-cell therapy, where T cells are genetically altered to express a cancer-specific antigen receptor. Braun et al. tested laboratory-generated CAR-T cells targeting either EBV gp350 or the B-cell marker, CD19, in EBV-positive BL-bearing mice. Results showed that CD19-CAR-T cells were better-tolerated by the animals relative to gp350-CAR-T cells, and only treatment with CD19-CAR-T cells resulted in a therapeutic response [108]. A subsequent study by a different group demonstrated the efficacy of independent gp350-CAR-T cells in an EBV-positive BL mouse model [109]. These data warrant further testing of both CD19-CAR-T- and gp350-CAR-T-cell therapies in EBV-positive BL. Finally, a retrospective study examined the efficacy of CAR-T-cell therapy in adults with R/R BL. Out of 25 analyzed patients, 2 had confirmed EBV infection. CAR-T-cell therapy initially improved patient responses (52% overall response rate), but the duration of response was sub-optimal [110]. The short observed duration of therapeutic response could potentially be attributed to the aggressive nature of BL. A multicenter, retrospective study of 31 R/R BL patients who received CD19-CAR-T-cell therapy demonstrated similar results [111]. Overall, CAR-T-cell therapy holds potential promise for use in BL, although retrospective and/or prospective studies focusing on EBV-positive disease specifically are severely lacking. Combining CAR-T-cell therapy with chemotherapy or other immunotherapies may improve response outcomes.

### 4.5. Diffuse Large B-Cell Lymphoma

Similar to GC, the EBV status is not always considered when testing biotherapies in DLBCL. A retrospective study showed that a CD19-CAR-T-cell therapy, called axicabtagene ciloleucel, was effective in DLBCL patients with an unknown EBV status who had previously failed an autologous stem cell transplant. Although the majority of these patients had refractory disease, the one-year progression-free survival following axicabtagene ciloleucel administration was 56% [112]. A subsequent prospective, multicenter, single-arm phase-II clinical trial tested axicabtagene ciloleucel as a first-line monotherapy in high-risk large B-cell lymphoma. Results were encouraging, with an 86% complete response rate and a three-year progression-free survival rate of 75% [113]. Another biotherapy that has been tested in DLBCL is polatuzumab vedotin. This therapy is an antibody–drug conjugate containing a compound called monomethyl auristatin E (MMAE). MMAE is highly effective against cancer cells by breaking down the cellular structure and inducing apoptosis, but it is also quite toxic. Therefore, MMAE is linked to a “delivering” molecule that selectively targets cancer cells, therefore bypassing toxicity. In polatuzumab vedotin, MMAE is linked to CD79b, a B-cell-specific marker. A double-blind, controlled, international phase-III clinical trial showed that, when combined with chemotherapy, polatuzumab vedotin significantly increased two-year progression-free survival as a first-line therapy compared to standard chemotherapy alone in DLBCL [114]. However, EBV positivity was not assessed in this study.

A relevant case report documented the treatment of EBV-positive DLBCL in an immunocompetent patient who had previously failed chemotherapy. The patient was first given tiririzumab, an anti-PD-1 therapy, and a partial response was observed. CD19-CAR-T-cell therapy was subsequently administered, and the patient achieved complete remission [115]. The success of this case study suggests that anti-PD-1 therapy in combination with CD19-CAR-T-cell therapy may be a viable option for patients with EBV-positive DLBCL who failed frontline therapy. However, it is unclear if the same response would be achieved in an individual who was immunosuppressed prior to treatment initiation. A later study reiterated the use of anti-PD-1 therapy in EBV-positive DLBCL in patients who did not respond to frontline chemotherapy. In this small retrospective study, patients were given tislelizumab (N = 2), sintilimab (N = 3), or camrelizumab (N = 1) in conjunction with chemotherapy. Four patients (N = 2 tislelizumab and N = 2 sintilimab) achieved a complete response; one patient (N = 1 sintilimab) achieved a partial response; and one patient (N = 1 camrelizumab) did not respond and progressed [116]. Together, these data support prospective testing of anti-PD-1 therapy in refractory EBV-positive DLBCL. Indeed, a phase-II clinical trial tested tislelizumab combined with zanubrutinib (a BTK inhibitor) in patients with R/R EBV-positive DLBCL (NCT04705129), and an open-label, single-center, non-randomized phase-II clinical trial tested sintilimab in combination with rituximab and chemotherapy as a frontline therapy for DLBCL (NCT04023916). However, results from these studies are not available to date.

### 4.6. NK/T-Cell Lymphoma

As NKTL is uniformly EBV-positive, therapies targeting EBV are an attractive candidate for this disease. LMP1 is a major EBV oncoprotein expressed in NKTL. Li et al. generated CAR-T cells that were specific for LMP1 and/or CD38, an immune molecule expressed in NKTL and previously reported to be a treatment target for multiple myeloma [117]. Infusions of control T cells, LMP1-CAR-T cells, CD38-CAR-T cells, or tandem CAR-T cells targeting both LMP1 and CD38 were given to NKTL-bearing mice. Results showed that tandem CAR-T cells were the most effective, followed by CD38-CAR-T cells. That said, all three therapies significantly reduced the tumor burden compared to the control T cells [118]. These data warrant further investigation into CD38-LMP1-CAR-T-cell therapy for NKTL.

While CAR-T-cell therapy involves genetically engineering a T-cell receptor, other T-cell therapies harness the patient’s existing T cells without modification. A multicenter phase-II clinical trial tested a novel biotherapeutic, called baltaleucel-T, for R/R NKTL [119]. Baltaleucel-T is a bioproduct made by taking whole blood from EBV-infected patients and exposing the antigen-presenting cells (APCs) to EBV-specific antigens. The patient’s T cells are then exposed to these EBV-APCs, activating and expanding the EBV-specific T-cell population. These EBV-specific T cells are then injected back into the patient. As this therapy takes time to manufacture, and NKTL is a very aggressive disease, many patients in this cohort were lost to disease progression prior to infusion and/or prior to treatment completion. However, in the remaining patients, the results were promising, as 30% of patients demonstrated a complete response and 20% of patients demonstrated a partial response [119]. Potential combination of baltaleucel-T with other agents, such as immune checkpoint inhibitors, might increase its efficacy, but future studies will be needed to test this proposal.

Brentuximab vedotin is a biotherapy that targets CD30, a molecule frequently found on EBV-positive lymphoma cells including NKTL, and contains an MMAE linker. A multicenter phase-II clinical trial (N = 25) found brentuximab vedotin to be effective in R/R NKTL, with an overall response rate of 46% and a complete response rate of 18% [120]. Limitations of this study include the lack of a diverse population (South Korean cohort only) and a male majority (80%). Additionally, this trial was conducted simultaneously on other lymphoma subtypes as well, so further testing of brentuximab vedotin in NKTL-specific cohorts is warranted.

Anti-PD-1 therapy is an emerging biotherapeutic in NKTL. In an R/R NKTL cohort in Korea (N = 59), treatment with pembrolizumab following one or several lines of failed chemotherapy resulted in a 41% overall response rate, with 29% of patients achieving a complete response [121]. Pembrolizumab is currently being tested in a Chinese multicenter, open-label, single-arm phase-II clinical trial in combination with radiotherapy as a frontline treatment for NKTL patients who cannot receive chemotherapy (NCT04417166). A retrospective study analyzed the success of sintilimab a small cohort of children with NKTL. Results showed that 66% of patients achieved a complete response (N = 2) and 33% achieved a partial response (N = 1) [122]. All patients had significant reductions in EBV DNA in both plasma and whole blood following anti-PD-1 therapy. Although promising, a major limitation of this study is the small sample size. Finally, sintilimab in combination with lenalidomide was tested in a single-center, prospective study for NKTL (N = 24). The overall response rate was found to be 54.2%, with 45.8% of patients achieving a complete response [123]. Additionally, EBV DNA in PBMCs significantly decreased and CD8+ T-cell numbers significantly increased following treatment. Interestingly, further analysis showed that levels of effective memory CD8+ T cells and interferon gamma were elevated in responders relative to non-responders after treatment [123], underscoring the importance of CD8+ T cells in this therapy. Sintilimab has also been tested in combination with chemotherapy in R/R NKTL. Results of this multicenter, single-arm, phase-II clinical trial were striking, demonstrating a 100% overall response rate and an 85% complete response rate [124]. Follow-up for this study is still ongoing (NCT04127227). Overall, these studies suggest that anti-PD-1 therapy is a safe and effective treatment modality for NKTL, especially in R/R patients who have exhausted available chemotherapies.

### 4.7. Post-Transplant Lymphoproliferative Disorder

R/R EBV-positive PTLD patients have few treatment options and a mean overall survival of less than five months, making development of new therapeutics for this disease essential. A third kind of T-cell therapy (allogeneic) involves cryobanking T cells from healthy donors and then matching these cells to patients based on HLA typing. For EBV-positive malignancies, these cryobanked T cells are taken from EBV seropositive individuals. An EBV-specific allogeneic biotherapy, called tabelecleucel, has been tested under various conditions in R/R EBV-positive PTLD, with promising results. Results of a multicenter, expanded access protocol demonstrated that tabelecleucel resulted in a 65.4% overall response rate in R/R patients and a two-year overall survival rate of 70% [125]. A global, multicenter phase-III clinical trial called the ALLELE trial tested tabelecleucel in EBV-positive PTLD patients who had failed rituximab or rituximab plus chemotherapy, and demonstrated a 51% response rate with a tolerable safety profile [126]. Updated results from the ALLELE trial confirmed the 51% overall response rate and revealed a median duration of response of 23 months and median overall survival of 18.4 months [127]. These results are striking and represent a significant improvement in patient prognoses compared to no treatment in R/R EBV-positive PTLD. To date, tabelecleucel has been approved for use in the European Union, but is still undergoing testing to gain FDA approval in the United States.

A similar allogeneic T-cell therapy, called LMP-TC, was tested in both newly diagnosed and R/R EBV-positive PTLD pediatric patients (<30 years of age) in a pilot phase-II clinical trial [128]. LMP-TC are T cells that are specific for the EBV latent membrane protein antigens, LMP1 and LMP2. In brief, both B and T cells are isolated from third-party EBV-seropositive donors. The donor B cells are immortalized with EBV and transduced with LMP expression vectors that co-express LMP1 and LMP2. Then, the LMP-expressing transformed B cells are co-cultured with matched donor T cells in the presence of IL-2 to produce LMP-specific CD8+ T cells. These EBV-specific T cells are then infused into patients based on HLA allele typing. In the newly diagnosed patient cohort (N = 10), the overall response rate was 70%, with 40% achieving a complete response. In the R/R cohort (N = 5), the overall response rate was 20%, with no patients achieving a complete response. Two-year overall survival rates were 92% in the newly diagnosed cohort and 83% in the R/R cohort [128]. Overall, this study demonstrated the utility of LMP-TC therapy in EBV-positive PTLD, with very favorable results observed in newly diagnosed patients. Therefore, these data support LMP-TC therapy as a potential frontline treatment for EBV-positive PTLD.

### 4.8. Discussion

A summary of the discussed biotherapies for EBV-positive malignancies can be found in Table 3. Some biotherapies could potentially be used in the future as first-line treatment, such as LMP1/2-specific T cells for EBV-positive PTLD, which demonstrated an overall response rate of 70% in newly diagnosed patients. Importantly, many of the discussed emerging biotherapies have elicited positive therapeutic responses in R/R disease, which remains a major hurdle in cancer treatment. Two biotherapy categories, immune checkpoint inhibition and T-cell-based therapy, have shown promise across different EBV-positive malignancies in this regard. PD-1 inhibitors improved the response rate in NPC compared to chemotherapy alone in both R/R and metastatic disease. Anti-PD-1 therapy combined with chemotherapy may also be useful in managing refractory EBV-positive GC. Additionally, PD-1 inhibitors have demonstrated efficacy in refractory NKTL and EBV-positive DLBCL, although in the latter, the sample sizes were small. Finally, anti-PD-1 monotherapy induced sustained responses in multiple cohorts of HL patients with R/R disease as well as in newly diagnosed patients, although the EBV status was not determined in these studies. In R/R EBV-positive PTLD, the allogeneic T-cell therapy, tabelecleucel, has shown safety and efficacy (>50% overall response rate) in multiple studies, and is currently licensed for use in the European Union following failed rituximab or immunochemotherapy. CD19-CAR-T-cell therapy has been evaluated in DLBCL with encouraging response rates, although the EBV status was not considered. Several CAR-T-cell-based therapies have also been tested in animal models of EBV-positive BL and NKTL. Both EBV-specific and immune cell-specific (CD19, CD38) T cells reduced the tumor burden, suggesting that tandem CAR-T cells targeting both EBV-expressing cells and immune cells may be most effective in these malignancies. However, challenges exist for developing effective CAR-T-cell therapies in BL, as the exceptionally aggressive nature of this cancer may shorten any conferred therapeutic benefit. Indeed, retrospective studies in pan-BL support this hypothesis, and therefore combining CAR-T-cell therapy with another intervention could be one strategy to improve outcomes.

## 5. Conclusions and Future Directions

While almost all adults are infected with EBV, only a small proportion of infected individuals develop EBV-associated cancers. The underlying environmental, genetic, and viral mechanisms of EBV oncogenesis are still being elucidated, but immunosuppression is one known factor, especially in diseases such as PTLD. The field has made great strides in identifying diagnostic and prognostic biomarkers for EBV-driven cancers and in developing novel biotherapies to help combat these notoriously difficult-to-treat malignancies. Across the carcinomas and lymphomas associated with EBV, plasma EBV DNA levels have emerged as a useful biomarker with both diagnostic and prognostic value. The methylation status of EBV DNA and certain EBV microRNAs (BARTs) have also been identified as potential biomarkers in these cancers. On the therapeutic side, biotherapies such as NK-cell therapy, CAR-T-cell therapy, immune checkpoint therapy (PD-1/PD-L1 inhibitors), and antibody drug conjugates have all demonstrated safety and efficacy in EBV-associated cancers. Importantly, many of these biotherapies are effective following the failure of first-line therapy, offering new options for patients with refractory disease and otherwise dismal prognoses.

While diagnostic markers aid in early detection of cancer, predictive biomarkers are present years before diagnosis and can help identify patients at high risk of developing cancer in the future. An example of a predictive biomarker is gp42-IgG titers in EBV-positive NPC. Patients with high levels of gp42-IgG had a 71% reduced risk of developing NPC, and this protective factor was present over five years prior to NPC diagnosis [129]. The identification of protective, predictive biomarkers for EBV-driven cancers can not only aid in monitoring high-risk patients (and thus enable early detection) but also identify therapeutic targets for the development of prophylactic vaccines (reviewed in [130]) or other biological interventions such as monoclonal antibodies. Earlier this year, Chhan et al. described the generation of EBV-specific human monoclonal antibodies, anti-gp350 and anti-gp42, which partially or completely protected humanized mice from EBV challenge, respectively [131]. As EBV infection is usually acquired early in life, a preventative EBV vaccine would most likely have to be given before or at birth to be effective. However, prophylactic vaccines or antibodies could be useful throughout life, especially in individuals who are at high risk of developing EBV-associated cancers. Overall, the future remains bright for immune-based therapies in malignancies associated with EBV infection.

## Figures and Tables

**Table 1:** EBV-associated malignancies and their characteristics.

Cancer Type	Disease	Immunogenicity * (Latency Stage)	First-Line Treatment
Carcinoma	Nasopharyngeal carcinoma	Moderate (II)	Chemoradiation
	Gastric cancer	Low–moderate (I–II)	Surgical resection
Lymphoma	Burkitt lymphoma	Low (I)	Chemotherapy plus rituximab ^#^
	Diffuse large B-cell lymphoma	Moderate–high (II–III)	Chemotherapy plus rituximab
	NK/T-cell lymphoma	Moderate (II)	Radiotherapy
	Hodgkin lymphoma	Moderate (II)	Chemotherapy
Lymphoproliferative disorder	Post-transplant	High (III)	Immunosuppressive drug reduction
	lymphoproliferative disorder		

* Based on viral gene expression. ^#^ Anti-CD20 antibody.

**Table 2:** Emerging biomarkers for EBV-associated cancers.

EBV-Associated Cancer	In Development	Being Tested in Clinical Trials	Currently Available for Use
NPC	EBNA1-IgA (outside Asia); BBLF4-L322M (East Asia only); miR-BART8-3p; methylated EBV DNA; PD-L1; PTEN	anti-BNLF2b (P85-Ab); PD-1/CTLA-4	EBNA1-IgA (Asia only); BALF2 162476T>C and 163364C>T (Asia only); plasma EBV DNA
GC	CTLA-4; TMB; SMARCA4; PD-L1; miR-BART6-5p, -BART2-5p, -BART7-3p, -BART13-3p, -BART1-5p, -BART4-5p, and -BART20-5p; CHAF1A	-	plasma EBV DNA
HL	-	-	plasma/whole-blood EBV DNA
DLBCL	EBV copy number (≥10^4^ copies/mL); low hemoglobin/anemia	-	plasma/whole-blood EBV DNA
NKTL	EBER; JAK3; monocyte levels	-	plasma EBV DNA
PTLD	sZEBRA; miR-BHRF1-1; miR-BART2-5p; methylated EBV DNA	-	plasma EBV DNA

NPC = nasopharyngeal carcinoma; GC = gastric cancer; HL = Hodgkin lymphoma; NHL = non-Hodgkin lymphoma; DLBCL = diffuse large B-cell lymphoma; NKTL = NK/T-cell lymphoma; PTLD = post-transplant lymphoproliferative disorder; miR = EBV microRNA; TMB = tumor mutational burden.

**Table 3:** Emerging biotherapies for EBV-associated cancers.

EBV-Associated Cancer	In Development	Being Tested in Clinical Trials	Currently Available for Use
NPC	NK-cell therapy + mRNA EBV vaccination	nivolumab; cadonilimab	tislelizumab
GC	anti-CD276	nivolumab	tislelizumab; pembrolizumab
HL	anti-LAG-3	ruxolitinib; AFM13-NK; tislelizumab	nivolumab; pembrolizumab
BL	anti-gp350/CD89; CD19-CAR-T; gp350-CAR-T	-	-
DLBCL	tiririzumab	tislelizumab + zanubrutinib; sintilimab	axicabtagene ciloleucel (CD19-CAR-T); polatuzumab vedotin
NKTL	CD38-CAR-T; LMP1-CAR-T	baltaleucel-T; brentuximab vedotin; pembrolizumab; sintilimab	-
PTLD	-	tabelecleucel (United States); LMP-TC	tabelecleucel (European Union)

NPC = nasopharyngeal carcinoma; GC = gastric cancer; HL = Hodgkin lymphoma; BL = Burkitt lymphoma; DLBCL = diffuse large B-cell lymphoma; NKTL = NK/T-cell lymphoma; PTLD = post-transplant lymphoproliferative disorder.

## Data Availability

No new data were created or analyzed in this study. Data sharing is not applicable to this article.

## Abbreviations

The following abbreviations are used in this manuscript:

APC	Antigen-presenting cell
BART	BamH1-A rightward transcript
BL	Burkitt lymphoma
BTK	Bruton’s tyrosine kinase
CAR	Chimeric antigen receptor
CCRT	Concurrent chemoradiotherapy
CHAF1A	Chromatin assembly factor 1 subunit A
CHL	Chronic high viral load
CTLA-4	Cytotoxic T lymphocyte-associated protein 4
DLBCL	Diffuse large B-cell lymphoma
DNA	Deoxyribonucleic acid
EBER	EBV-encoded RNA
EBNA1	Epstein–Barr nuclear antigen 1
EBV	Epstein–Barr virus
GC	Gastric cancer
HIV	Human immunodeficiency virus
HL	Hodgkin lymphoma
ISH	In situ hybridization
JAK	Janus kinase
LAG-3	Lymphocyte activation gene 3
LMP	Latent membrane protein
mIR	MicroRNA
MMAE	Monomethyl auristatin E
mRNA	Messenger RNA
NHL	Non-Hodgkin lymphoma
NKTL	NK/T-cell lymphoma
NPC	Nasopharyngeal carcinoma
PBMC	Peripheral blood mononuclear cell
PD-1	Programmed cell death protein 1
PD-L1	Programmed death ligand 1
PTEN	Phosphatase and tensin homolog
PTLD	Post-transplant lymphoproliferative disorder
R/R	Relapsed or refractory
RNA	Ribonucleic acid
SMARCA4	SWI/SNF-related matrix-associated actin-dependent regulator of chromatin subfamily A, member 4
SNP	Single-nucleotide polymorphism
sZEBRA	Soluble form of the EBV ZEBRA protein
TIL	Tumor-infiltrating lymphocyte
TMB	Tumor mutational burden
